# Interaural and sex differences in the natural evolution of hearing levels in pre-symptomatic and symptomatic carriers of the p.Pro51Ser variant in the *COCH* gene

**DOI:** 10.1038/s41598-023-50583-6

**Published:** 2024-01-02

**Authors:** Julie Moyaert, Annick Gilles, Griet Mertens, Marc J. W. Lammers, Hanne Gommeren, Sebastien Janssens de Varebeke, Erik Fransen, Nicolas Verhaert, Sam Denys, Raymond van de Berg, Ronald Pennings, Olivier Vanderveken, Vincent Van Rompaey

**Affiliations:** 1 Department of Translational Neurosciences, Faculty of Medicine and Health Sciences, University of Antwerp, Antwerp, Belgium; 2 Department of Otorhinolaryngology and Head and Neck Surgery, Antwerp University Hospital, Edegem, Belgium; 3 Department of Otorhinolaryngology and Head and Neck Surgery, Jessa Hospital, Hasselt, Belgium; 4 Centre of Medical Genetics, University of Antwerp, Antwerp, Belgium; 5 Department of Neurosciences, Research Group Experimental Otorhinolaryngology (ExpORL), KU Leuven, Leuven, Belgium; 6 Department of Otorhinolaryngology and Head and Neck Surgery, University Hospitals of Leuven, Leuven, Belgium; 7 Division of Balance Disorders, Department of Otorhinolaryngology and Head and Neck Surgery, Faculty of Health Medicine and Life Sciences, Maastricht University Medical Center, Maastricht, The Netherlands; 8 Department of Otorhinolaryngology and Head and Neck Surgery, Radboud UMC, Nijmegen, The Netherlands

**Keywords:** Genetics research, Diagnosis

## Abstract

Hearing impairment constitutes a significant health problem in developed countries. If hearing loss is slowly progressive, the first signs may not be noticed in time, or remain untreated until the moment the auditory dysfunction becomes more apparent. The present study will focus on DFNA9, an autosomal dominant disorder caused by pathogenic variants in the *COCH* gene. Although several cross-sectional studies on this topic have been conducted, a crucial need for longitudinal research has been reported by many authors. Longitudinal trajectories of individual hearing thresholds were established as function of age and superimposed lowess curves were generated for 101 female and male carriers of the p.Pro51Ser variant. The average number of times patients have been tested was 2.49 years with a minimum of 1 year and a maximum of 4 years. In addition, interaural and sex differences were studied, as they could modify the natural evolution of the hearing function. The current study demonstrates that, both in female carriers and male carriers, the first signs of hearing decline, i.e. hearing thresholds of 20 dB HL, become apparent as early as the 3rd decade in the highest frequencies. In addition, a rapid progression of SNHL occurs between 40 and 50 years of age. Differences between male and female carriers in the progression of hearing loss are most obvious between the age of 50 and 65 years. Furthermore, interaural discrepancies also manifest from the age of 50 years onwards. High-quality prospective data on the long-term natural evolution of hearing levels offer the opportunity to identify different disease stages in each cochlea and different types of evolution. This will provide more insights in the window of opportunity for future therapeutic intervention trials.

## Introduction

Hearing impairment is one of the most frequent types of sensory deficits in the human population. The World Health Organization (WHO) estimates that, by 2050, approximately 2.5 billion people will have hearing loss, and therefore it has been recognized as a priority condition for research to find therapeutic solutions^1^. Currently, a treatment to prevent or stop hearing deterioration in patients with sensorineural hearing loss (SNHL) is not available. Previous studies have demonstrated that hearing loss is correlated with decreased quality of life and lower psychological well-being^2–5^. Accordingly, there is a fourfold increase in symptoms of anxiety and depression in patients with hearing loss compared with the general population^6–8^. Furthermore, it was shown that decreased hearing levels correlate with lower cognitive performance^9–11^. Consequently, hearing loss was identified as an independent modifiable risk factor for accelerated cognitive decline, cognitive impairment and even dementia in older adults^12–15^. The Lancet Commission declared that, if mid-life hearing loss could be precluded, the risk of dementia might be reduced by nine percent^16^. As such, the global burden of hearing impairment induces a leading health problem^17^ and it is of paramount importance to focus on the starting point of hearing deterioration. If hearing loss is slowly progressive, the first signs can be easily overlooked, and no treatment or rehabilitation will be initiated until the moment the auditory deficits result in limitations in daily life activities.

The present study focuses on DeaFNess Autosomal 9 (DFNA9), an autosomal dominantly inherited inner ear disorder (ORPHA: 90635), caused by pathogenic variants in the Coagulation Factor C Homology (*COCH*) gene. This pathogenic variant (previously called a mutation) induces a progressive decline in hearing and vestibular function and will, eventually, lead to post-lingual severe-to-profound SNHL and vestibular impairment^18–20^. In Belgium and the Netherlands, it is estimated that about 1000 people are carriers of this pathogenic variant, making it one of the most frequent types of hereditary, age-related SNHL in this region^20^. Over 30 different heterozygous *COCH* gene variants have been identified worldwide. The *COCH* gene has 12 exons that encode the secreted protein cochlin, which is expressed at high levels in the inner ear, specifically in the fibrocytes of the spiral ligament and spiral limbus^21^. As known, various pathogenic variants are associated with the hearing impairment, leading to DFNA9. However, the pathophysiology of DFNA9 is not yet fully understood, but several mechanisms have been suggested and may be present at different stages. These are: protein misfolding leading to cytotoxic cochlin aggregates and fibrocyte degeneration^22,23^; reduced LCCL secretion into the extracellular matrix (ECM), leading to a reduced anti-inflammatory response and accumulation of endotoxin^24^; reduced cleavage of LCCL by aggrecanase leading to a reduced concentration of LCCL peptides in the perilymph of the scala tympani needed for antibacterial activity^25^; degradation of ECM, resulting in dendritic and neuroepithelial cell death^26,27^. This neuronal degeneration has been observed in histological specimens obtained in patients with DFNA9^28^.

The majority of patients in Belgium and The Netherlands are carriers of the c.151C>T variant example in the LCCL (limulus factor C, cochlin, late gestation lung protein Lgl1) domain of the cochlin gene (COCH), resulting in a replacement of amino acid Proline, at position 51**,** by Serine (p.Pro51Ser)^29^. Moreover, the actual position of this identified variant is 14-30877640-C-T (GRCh38) and the reference sequence for c.151C>T is annotated on transcript NM_001135058.1^30^. As a result, a series of genotype–phenotype correlation studies in families with DFNA9 have previously been published. The majority of these studies have presented retrospective and prospective cross-sectional data which indicated a late-onset, progressive, sensorineural hearing loss^29,31–39^.

Comprehensive longitudinal designs to assess the natural course of the P51S phenotype and therefore offering a better understanding of the degree of change over time, are lacking^40^. Understanding the natural evolution, however, is essential to predict the age of onset and to find the critical period of the most significant functional deterioration in patients with DFNA9. After all, the optimal therapeutic approach would be to prevent the onset of the pathophysiology or prevent its progression in the pre-symptomatic or early symptomatic stage of the disease respectively^41^. In addition, interaural and sex-related differences in cochleovestibular dysfunction has remained largely unexplored in patients with DFNA9. As such, the primary objective of the current study is to visualize the longitudinal trajectories of hearing level as a function of baseline age in pre-symptomatic and symptomatic carriers of the p.Pro51Ser variant in the *COCH* gene. This prospective longitudinal study design will investigate the baseline age window at which hearing deterioration starts and progresses in the DNA9 population. The second objective is to examine how interaural and sex differences could modify the longitudinal trajectories of hearing function.

## Methodology

### Study design

In this prospective longitudinal multicenter study, recruitment took place between February 2019 and September 2022 at the Department of Otorhinolaryngology-Head and Neck Surgery at the Antwerp University Hospital (UZA), Jessa Hospital in Hasselt and the Leuven University Hospital. The study was designed and conducted according to the Declaration of Helsinki (1996) and approved by the local ethics committees of the Antwerp University Hospital (B300202042807) and UZ Leuven. The protocol of the study was conducted in a single session by an ICH-GCP-accredited clinical researcher and Master in Audiology. Execution of the protocol occurred annually for each patient in the same conditions. Prior to the start of the study, an informed consent was obtained from all study participants. All data (results of the audiometric assessment and questionnaires) have been stored in OpenClinica, a password-protected online database for data storage and processing.

### Enrollment, inclusion and exclusion criteria

**Figure 1:**
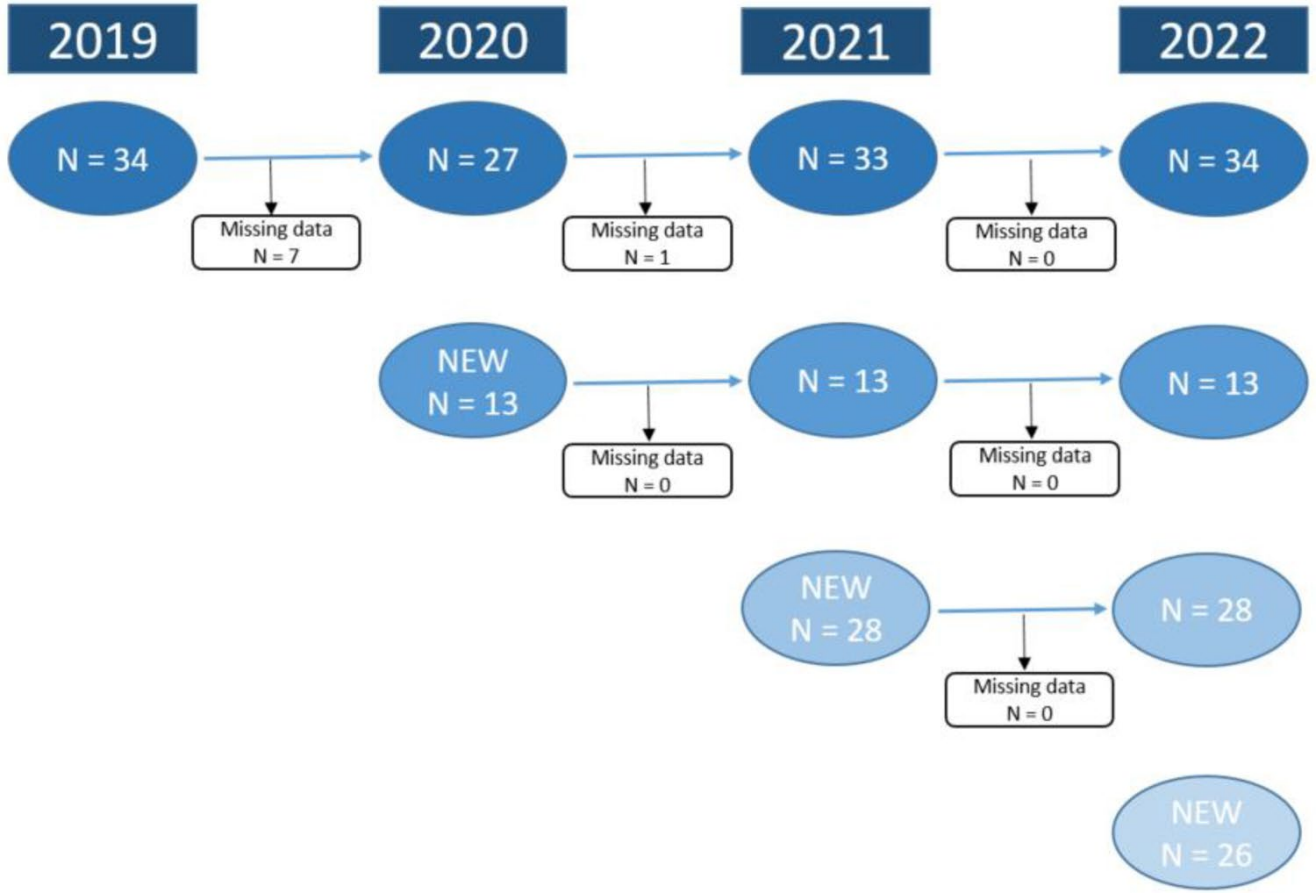
Inclusion of pre-symptomatic and symptomatic carriers of the p.Pro51Ser variant in the COCH gene.

All patients were diagnosed with DFNA9 through blood samples for genetic diagnosis as DNA analysis was executed through the sequencing of exon 4 (LCCL domain) of the COCH gene. The p.Pro51Ser variant in the *COCH* gene had to be documented, and all carriers aged 18 years or older were regarded as eligible for the study. No upper age limit was designated. Subsequently, during the first study visit, an interview with the patient was conducted to determine eligibility and to check inclusion and exclusion criteria. Exclusion criteria were active middle ear conditions at the time of first testing resulting in a conductive hearing loss with an air–bone gap exceeding 15 dB hearing level (HL) [i.e. a difference of at least 15 dB (HL) between air and bone conduction at three subsequent frequencies]^42^, SNHL or vestibular dysfunction due to other causes than DFNA9, known neurological and central nervous system disorders, intracranial disease/tumor, and not willing or not able to attend the yearly study visits. Recruitment and testing of the participants is depicted in Fig. 1 as inclusion started at the beginning of 2019 in UZA, Jessa hospital in Hasselt and the Leuven University Hospital. In the consecutive years, these patients were re-evaluated and every year new patients were added to the study. The study is still ongoing but to date, a total number of 101 patients with the p.Pro51Ser variant in the *COCH* gene were tested in 2022.

### Evaluation of hearing level

Pure-tone audiometry was conducted to evaluate unaided hearing levels. In a soundproof booth, air conduction thresholds were first determined using a two-channel Interacoustics AC-40 audiometer and headphones. Hearing level (expressed in dB HL) at frequencies of 125, 250, 500, 1000, 2000, 3000, 4000, 6000 and 8000 Hz were tested in both ears. Subsequently, thresholds for bone conduction were tested with the bone conductor in the frequency-range of 250–4000 Hz. The Hughson–Westlake methodology was followed and appropriate masking was applied when required. When a subject was unable to hear a tone, the highest audiometer output level was recorded as the threshold (i.e., 120 dB HL). Testing of hearing sensitivity happened blindly, without consulting previous results when they were available. An illustrative example of the evaluation of hearing level, at different timepoints, is depicted in Supplementary Fig. 1.

### Statistical analysis

Statistical analyses were carried out using the statistical software package R, version 4.1.2. (R: a language and environment for statistical computing. R Foundation for Statistical Computing, Vienna, Austria, 2021). The data assembled in this study constitutes of a cross-sectional and longitudinal component, covering a wide age-range in a short study duration. More specifically, baseline age of the participants ranged from 18 to 75 years and patients contributed with a variable length of follow up, with a maximum of four consecutive years. Therefore, individual responses of hearing level per frequency as function of age were visualized using spaghetti plots, showing an individual line for each subject. The spaghetti plots were generated using the ggplot2 package (H. Wickham. ggplot2: Elegant Graphics for Data Analysis. Springer-Verlag New York, 2016). To investigate age and sex effects, the group mean rates of change for males and females were drawn as a lowess line and added to the plots. This allows the comparison of age-related changes between males and females. Similarly, spaghetti plots of the individual patients’ difference in hearing thresholds of the left ear and right ear per frequency were plotted over time, to examine the interaural differences in the evolution of hearing level.

### Ethics approval

The study was designed and conducted according to the Declaration of Helsinki (1996) and the study protocol was reviewed and approved by the local ethics committees of the Antwerp University Hospital (B300202042807) and UZ Leuven.

### Informed consent

Informed consent was obtained from all individual participants included in the study. Participants signed informed consent regarding publishing their data.

## Results

### Demographics

Demographic details of age and sex were determined for the total study population of 101 patients at the time of the first study visit. Hence, audiological thresholds were documented in 202 ears. An overview of the demographic data can be found in Table 1. The average age in this DFNA9 population was 43.62 years, with a minimum of 18 years and a maximum of 67 years. Within this study group, 53% were female, and 47% were male. The average number of times patients have been tested in this prospective longitudinal study was 2.49 years with a minimum of 1 year and a maximum of 4 years.

**Table 1:** Demographic details of the study population, all carriers of the P51S variant in the COCH gene at the time of the first study visit (n = 101).

Age (decade)	Age (mean) ± SD	Age (range)	Number (N)	Number male (N)	Number female (N)
Third	22.0 ± 2.85	18–27	14	5	9
Fourth	35.91 ± 2.97	30–39	22	11	11
Fifth	44.54 ± 2.93	40–49	28	16	12
Sixth	53.39 ± 2.64	50–59	28	10	18
Seventh	62.89 ± 2.18	60–67	9	5	4
Total	43.62 ± 12.06	18–67	101	47	54

### Prospective natural evolution of hearing level

**Figure 2:**
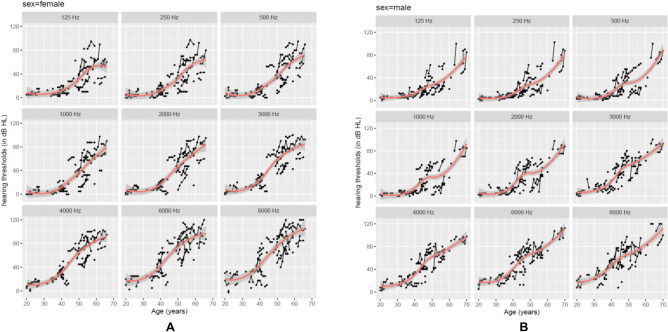
(**A**,**B**) Prospective natural evolution of hearing level. Cross-sectional data (in dots) and longitudinal trajectories (in solid black lines) of individual hearing thresholds (in dB HL) as a function of age for separate frequencies (125–8000 Hz). The red line curve is a superimposed lowess curve across the entire age span. (**A**) The individual performance and mean function per frequency over time for female carriers of the P51S variant, whereas (**B**) shows the male carriers of the P51S variant.

To demonstrate the longitudinal trajectories of an individual patient’s hearing thresholds within the actual follow-up window, individual dots and lines were drawn. Figure 2A presents the plots for female carriers and Fig. 2B for male carriers. Hearing thresholds as a function of age are shown for every frequency. Superimposed lowess curves were calculated as a nonparametric estimator of the overall evolution of hearing thresholds over time.

In general, hearing deterioration evolves differently depending on the frequency. In female carriers (Fig. 2A), the first signs of hearing loss (at least 20 dB HL) become apparent as early as in the 3rd decade for the highest frequencies (6 and 8 kHz). In addition, the graphs show a hearing threshold of 20 dB HL in the mid frequency (of 2 kHz) around the age of 40 years and in the low frequencies (below 1 kHz) at the age of 45 years. A hearing loss of at least 40 dB HL is estimated in the high frequencies of 6 and 8 kHz at the age of 40 years. In comparison, for the frequencies below 1 kHz, hearing thresholds of 40 dB HL and more severe are only apparent from the age of 50–55 years. As hearing loss is progressive, hearing thresholds exceeding 70 dB HL are estimated at 50 years of age at the frequency of 4 kHz. For higher frequencies, this hearing loss even starts in patients younger than 50 years. In the mid frequency range (2 and 3 kHz), hearing thresholds of at least 70 dB HL become apparent between 55 and 60 years of age.

In male carriers (Fig. 2B), hearing loss of at least 20 dB HL also starts at the age of 30 years for the highest frequencies (6 and 8 kHz). Similar to female carriers, hearing thresholds of 20 dB HL can be found at 40 years of age in the mid frequency (of 2 kHz) and 45 years of age for the low frequencies (below 1 kHz). Progressively, hearing decline of 40 dB HL in the highest frequencies become apparent from the early age of 40 years. Hearing thresholds of 40 dB HL are estimated at the age of 45 years in the mid frequencies and 55–60 years in the low frequencies. Hearing loss exceeding 70 dB HL was expected at the age of 55 years in the high frequencies, at 55–60 years in the mid frequencies (3 and 4 kHz) and at 65–70 years in the low frequencies (< 2 kHz).

### Impact of sex on hearing loss progression

**Figure 3:**
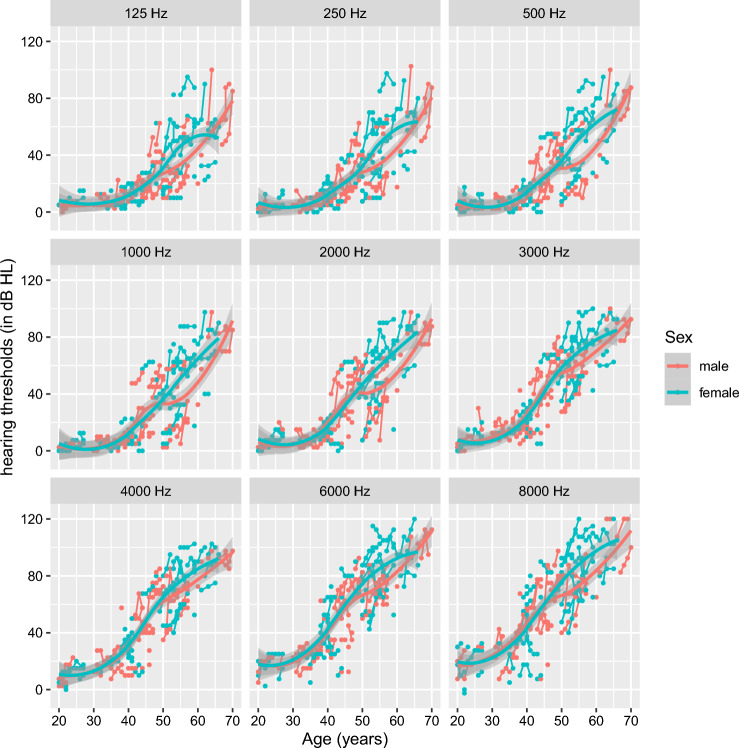
Impact of sex on evolution of hearing loss. combination of Fig. 2A,B whereas orange lines represent all data of male carriers and blue lines show all data of female carriers of the P51S variant as a function of time.

In Fig. 3, the graphs in Fig. 2A,B are merged to visualize sex-related differences in hearing loss progression. Differences in hearing deterioration between males and females younger than 40 years are limited across all frequencies. The progression of hearing levels from normal hearing to mild hearing loss of 30 dB HL is similar in male and female carriers. However, differences can be observed between the age of 50 and 65 years. More specifically, while the pace of hearing loss progression is stable in female patients, the pace appears to slow down in the male population by the age of 50 years and accelerates again at 55 years. In addition, this difference seems to be more pronounced in the low frequencies up to -and including- 2 kHz compared to the mid and high frequencies (> 2 kHz).

### Interaural differences in the natural evolution of hearing level

**Figure 4:**
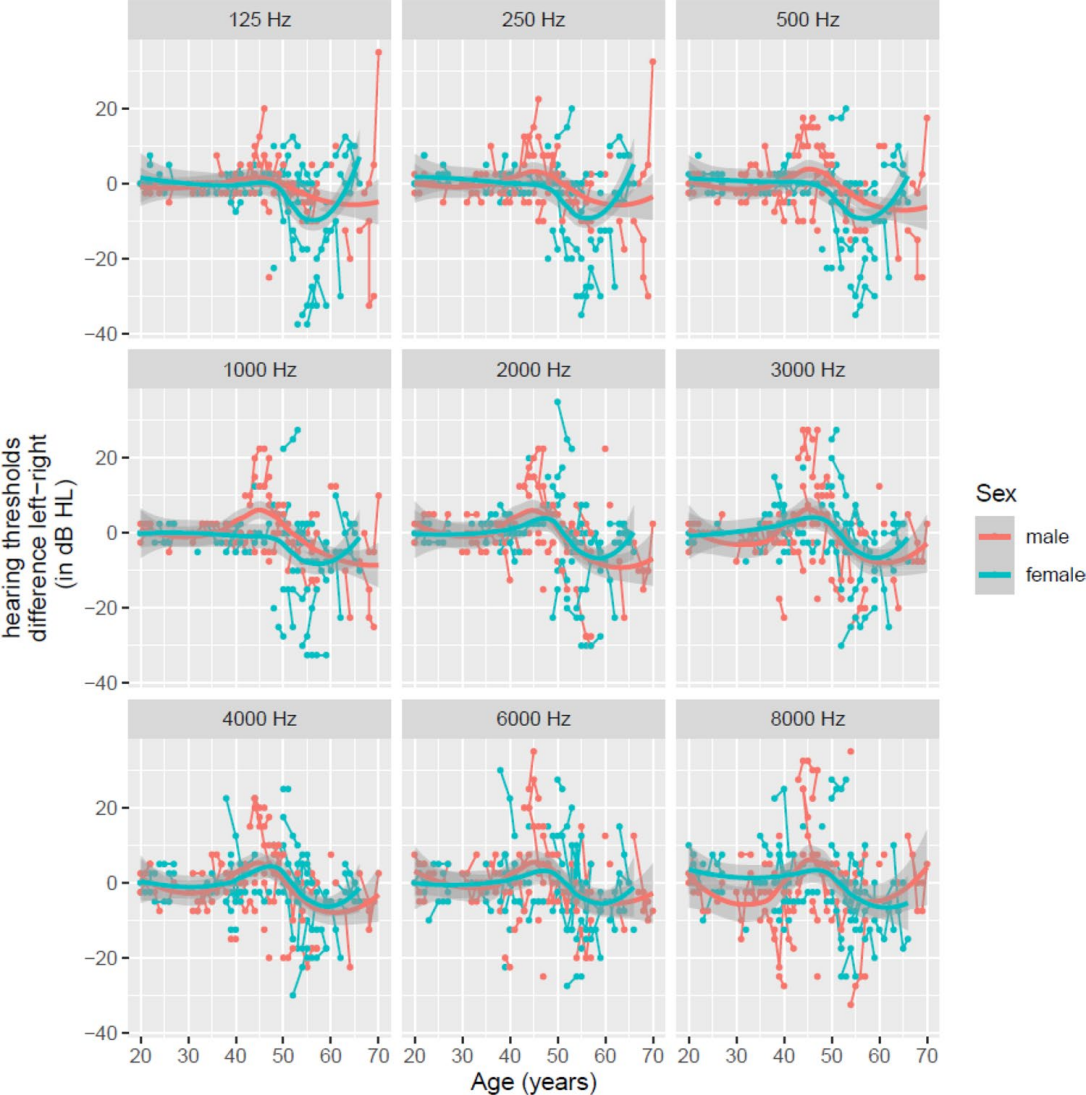
Interaural difference in hearing level. Every curve represents the individual patients hearing threshold of the left ear minus the right ear per frequency, over time, to investigate the interaural differences in the evolution of hearing level.

To study the interaural differences in the evolution of hearing level, Fig. 4 shows the difference in hearing thresholds per frequency (left ear threshold minus right ear threshold) as a function of age between 20 and 70 years. Similar to the study of Janssens de Varebeke et al., a difference of 10 dB HL between right and left ear thresholds is considered an acceptable test–retest difference^18^. No interaural difference was observed for male and female carriers between 18 and 50 years for all frequencies. Subsequently, more distinct differences in hearing thresholds become apparent, starting from the age of 50 years. The graphs imply that in the low frequencies (125–250 Hz) interaural differences become visible between 50 and 65 years of age. Furthermore, these differences are more pronounced in female carriers compared to male carriers. For the mid frequencies (2–4 kHz) the interaural difference tends to be present from the age of 55 years, however no differences were observed between males and females. In the high frequencies, interaural differences do not exceed 10 dB HL.

## Discussion

Longitudinal trajectories of individual hearing thresholds were investigated as a function of age and superimposed lowess curves were generated for female and male carriers of the p.Pro51Ser variant. Consistent with previous longitudinal studies, the current study confirms that SNHL is progressive as initially, at young age, the audio-profile is down-sloping, with preserved hearing at low and mid frequencies^33,34,36–39^. With advancing age, low and mid-frequency SNHL evolves and the audio-profile flattens^33^. Subsequently, hearing thresholds progress from moderate to severe hearing loss that eventually becomes profound across all frequencies between the age of 60 and 84 years old^20^.

In 2001, Verhagen et al. conducted a follow-up study of a family with DFNA9 and reported a mean deterioration of hearing thresholds of 3.8 dB per year in a variable, often asymmetric, pattern^37^. However, no frequency-specific information was provided. In contrast, the study of Lemaire et al.^39^ estimated a longitudinal annual threshold deterioration (ATD) of 2–3 dB/year at frequencies 250 Hz to 1 kHz, and an ATD of 3–5 dB/year for frequencies of 2–8 kHz. Nevertheless, this study included very limited longitudinal observations, with only five cases. A larger study of Bischoff et al.^34^, which included 20 patients, established that the annual threshold deterioration ranged from 1.9 dB/year to 3.3 dB/year (in the frequency-range of 0.5–8 kHz). The current study demonstrates that, both in female carriers and male carriers, the first signs of hearing decline, i.e. hearing thresholds of 20 dB HL, become apparent as early as in the 3rd decade of life in the highest frequencies (6 and 8 kHz). In female carriers, these findings confirm earlier cross-sectional data. However, in male carriers our findings differ from previous data of Janssens de Varebeke et al.^18^. In the latter study, hearing deterioration in male carriers started at about 46 years of age (fifth decade) at the highest frequency and the starting point of all the frequencies below 4 kHz was estimated in the fifth decade^18^. In that study, only one male subject was tested in the third decade of life and thus the contribution of (very) young male patients was therefore limited^18^, potentially explaining differences in study findings. In addition, one has to bear in mind that the differences in onset of hearing loss as a function of sex can be explained due to the differences between males and females in the ISO standards of hearing thresholds as a function of age. The main reason why the predictive values for onset of hearing loss for men have shifted so much compared to female carriers was due to the fact that these standards are stricter in males than in females, especially for the youngest generations^18^. Furthermore, hearing loss in DFNA9 patients is progressive and hearing decline of at least 40 dB HL seems to be present between 40 and 55 years in female carriers and between 40 and 60 years in male carriers. This supports previous cross-sectional data, in which a hearing decline of 40 dB HL was observed at 48 years, on average, in female carries and 50 years, on average, in male carriers. Similarly, the current study revealed hearing loss exceeding 70 dB HL between 50 and 60 years for females and between 55 and 70 years for males. The study of Janssens de Varebeke et al.^18^ estimated a hearing decline to 70 dB HL around the age of 56 years and 59 years, respectively for female and male carriers. Furthermore, a phenotype-genotype study on autosomal dominant nonsyndromic hearing loss related to COCH was conducted by Oh et al.^43^. Their findings paralleled ours, showing that patients in their third decade of life exhibit hearing loss only in the highest frequencies, and that hearing deterioration commences at age 35. In addition, patients aged 40–59 exhibit a significant decrease in hearing ability^43^.

Hearing is also affected by presbyacusis which, in nature, is also progressive as this hearing loss begins in the high frequencies and shifts towards the lower frequencies with increasing age^44–46^. According to the ISO-7029 standard in 2004, the first signs of age-related hearing loss in females become apparent around 60 years, specifically in the middle and high frequencies^47^. In males, hearing loss of 20 dB HL started around 50 years in the highest frequency and 60 years in the mid frequencies (3 and 4 kHz)^48^. In a large study by Pearson et al.^49^ about gender differences in a longitudinal study of age-related hearing loss, the most significant sex differences with respect to hearing loss occurred around the age of 50 years in the frequency range of 3–8 kHz where the change in men was 10 dB per decade faster compared to women.

A difference in sex in carriers of the p.Pro51Ser variant was first described in the study of Bom et al.^36^ as estimated values of the ATD were 0.7 dB/year for women and 1.4 dB/year for men at the frequency of 4 kHz. Respectively, in the higher frequency of 8 kHz, a difference in ATD of 1.3 dB/year and 1.8 dB/year was estimated for female and male carriers. More recently, the cross-sectional study of Janssens de Varebeke^18^ described an annual hearing threshold deterioration of 2.7 dB/year and 3 dB/year on average for male and female carriers, respectively. As the calculation of the ATD is derived from the s-shaped regression line (intercept and slope) and not subjected to the differences in ISO standards, this could be markedly different between male and female carriers. As previously suggested with the relationship between ATD and sex, in the current study, the graphs imply an effect of sex in the pace of hearing decline as illustrated by different longitudinal trajectories of hearing function. More specifically, our results suggest that differences in the progression of hearing loss between male and female carriers are most obvious between the age of 50 and 65 years. The pace in male carriers first slows down and then accelerates, specifically in the low frequencies up to—and including—2 kHz. In the future, more research is needed to study the differences in evolution of hearing loss between the two groups, as a different screening method within a certain age group could be more appropriate to identify the onset of hearing impairment, i.e. the start of the (early) symptomatic stage.

Additionally, in patients with the p.Pro51Ser variant, a significant inter-subject and interaural variability in the evolution of pure-tone thresholds with increasing age was identified in numerous studies^34,36,37,39^. With respect to the interaural differences in the natural evolution of hearing level, our results demonstrate more distinct differences in hearing thresholds, starting from the age of 50 years onwards. These findings correspond with the study by Janssens de Varebeke et al. in which was concluded that interaural differences in hearing thresholds are limited in patients aged 18 to 39 years^18^. Nevertheless, differences between the right and left ear exceed 10 dB HL in the age group ranging from 40 to 59 years^18^. In addition, asymmetric hearing was also identified in the study by Verstreken et al., as 22 of the 60 patients with DFNA9 had asymmetric hearing without any preference for ear^38^. Studies regarding left–right asymmetry in hearing thresholds levels in the general population are scarce. One study of Pirilä et al.^50^ found an average inferiority of the left ear, specifically in the frequently range of 3–6 kHz among young and adult subject. In addition, this inferiority was considerably smaller among females in comparison with males.

The sex and interaural differences revealed in the current study provide relevant new data that can support optimal hearing rehabilitation, e.g. by conventional hearing aids or cochlear implantation. However, one has to bear in mind that speech perception in patients with DFNA9 may evolve differently than liminal audiometry, which can impact hearing rehabilitation significantly. Hence, future research should focus on investigating speech perception in quiet (SPIQ) and noise (SPIN) in patients with the p.Pro51Ser variant. This could provide valuable information regarding the period in which the pathophysiology can lead to a worse SPIN than expected based on pure-tone hearing thresholds. In the pre-symptomatic stage, SPIN could be impaired before the onset of hearing loss on pure-tone audiometry. In addition, SPIN could be affected more significantly than expected in symptomatic subjects that already have SNHL. This discrepancy may provide insight in early disease stages that reflect synaptopathy or spiral ganglion neuron degeneration^51^.

In conclusion, prospective longitudinal data confirms the progressive loss of hearing function for all frequencies in both female and male carriers of the p.Pro51Ser variant. More specifically, a rapid progression of SNHL occurs between 40 and 50 years of age in both sexes. In addition, a sex-biased effect in the pace of hearing decline seems to be present as the pace in female carriers accelerates from the age of 50 years. Because the hearing deterioration endures throughout the entire life span of the patient, a longitudinal follow-up of, currently, maximum 4 years is still relatively limited. In addition, advanced statistical analyses are not yet possible due to the limited number of data points. Efforts will be made to ensure the long-term follow-up to at least 10 years. The current study, however, is the first to perform a longitudinal follow-up in a larger DFNA9 patient population in order to assess the precipitating effects of the disease on hearing, already providing important information on the hearing outcomes in DFNA9 patients. More high-quality prospective data on the long-term natural evolution of hearing levels offer the opportunity to identify different disease stages in each cochlea and different patterns of evolution (progressive course versus relapsing–remitting course). This will provide more insights in the window of opportunity for future therapeutic interventional trials^52^.

### Supplementary Information

Supplementary Information.

## Data Availability

The datasets used and/or analyzed during the current study are available from the corresponding author on reasonable request.
