# Supplementary material for: Interaural and sex differences in the natural evolution of hearing levels in pre-symptomatic and symptomatic carriers of the p.Pro51Ser variant in the COCH gene
Source: Sci Rep. 2024 Jan 2;14:184. doi: 10.1038/s41598-023-50583-6 (PMC10762206; doi:10.1038/s41598-023-50583-6)
Supplement: Supplementary file 1 — Supplementary Information. [file 41598_2023_50583_MOESM1_ESM.docx]

**Interaural and sex differences in the natural evolution of hearing levels in pre-symptomatic and symptomatic carriers of the p.Pro51Ser variant in the *COCH* gene**

Corresponding author

Julie Moyaert
[Julie.moyaert@uza.be](mailto:Julie.moyaert@uza.be)
ORCHID: 0000-0002-3383-5421


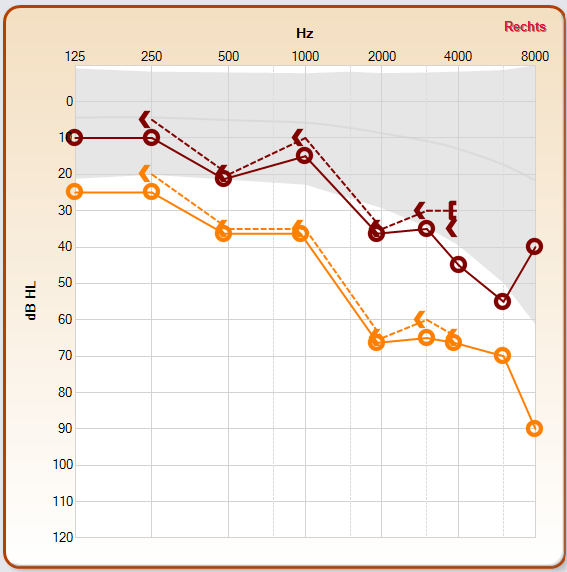

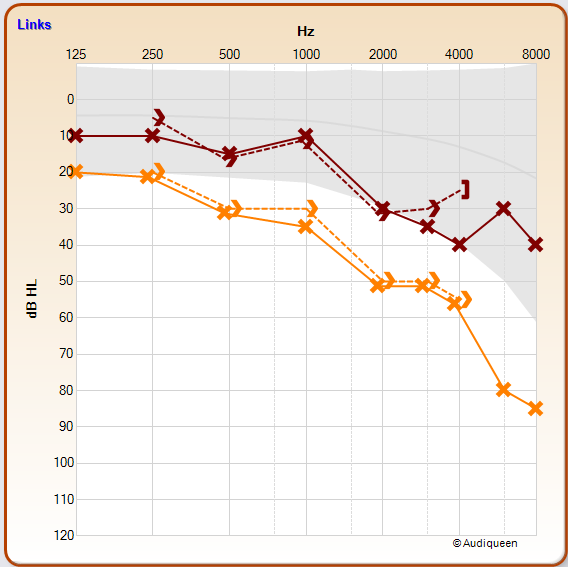


*Supplementary figure 1: an illustrative example of the evaluation of hearing level, at different timepoints.*
